# The Effect of the Addition of Fiber Preparations on the Color of Medium-Grounded Pasteurized and Sterilized Model Canned Meat Products

**DOI:** 10.3390/molecules26082247

**Published:** 2021-04-13

**Authors:** Mirosław Słowiński, Joanna Miazek, Krzysztof Dasiewicz, Marta Chmiel

**Affiliations:** Division of Meat Technology, Department of Food Technology and Food Evaluation, Institute of Food Sciences, Warsaw University of Life Sciences-SGGW, 02-787 Warsaw, Poland; miroslaw_slowinski@sggw.edu.pl (M.S.); joanna-miazek@wp.pl (J.M.); krzysztof_dasiewicz@sggw.edu.pl (K.D.)

**Keywords:** fiber preparations, canned meat products, pasteurization, sterilization, color

## Abstract

A beneficial aspect of the use of fiber preparations in the meat industry is the improvement of some quality characteristics of meat products. However, the preparation added in the amount of 3 or 6% may affect their color. The effect of the addition of barley, wheat and oat fiber preparations with different fiber lengths, in quantities allowing the product to be indicated as “high in fiber” or “source of fiber”, to pasteurized or sterilized medium-grounded canned meat products on their color, was determined. In the obtained canned meat products, the basic chemical composition and the L*, a* and b*, C* (Chroma) and h* (hue angle) color components were determined. The addition of the barley fiber preparation BG 300 to the model canned meat products caused a significant (*p* ≤ 0.05) darkening and an increase in the proportion of yellow color. In an industrial practice, this may result in poorer consumer acceptance of the meat product. Fiber length of wheat and barley fiber had no effect on the color components of products. The 6% addition of the wheat fiber preparations WF 200R and WF 600R or the oat fiber preparations HF 200 and HF 600 caused an apparent lightening of their color (ΔE > 2) compared to the control products.

## 1. Introduction

Dietary fiber is traditionally divided into two major groups: soluble and insoluble fiber [1,2,3]. Cereal-based fiber preparations can be obtained from the supporting parts of plants and contain mainly insoluble fiber (cellulose, hemicellulose, lignin), while those obtained from grains are rich in soluble fractions [4,5,6]. Wheat fiber, very popular in the food industry, belongs to the first group. It consists of almost 100% insoluble fiber fraction, so its nutritional value is low, and it is mainly a ballast component. Oat and barley fiber preparations, extracted from their grains which makes them rich in β-glucan, also deserve special attention. The content of β-glucan in barley is 2–8%, and in some, the so-called waxy varieties, its amount can reach 15% [7,8]. This component is the soluble fraction of dietary fiber, considered by some to be the greatest discovery of recent years [9]. Dietary fiber is a very important component of the human diet [1,4,10]. However, as a result of many changes in dietary patterns, most people in highly developed countries experience its deficiency [6]. Consumer awareness of the benefits of fiber consumption continues to increase. One way to increase intake and alleviate the effects of fiber deficiency is to incorporate fiber into meat products [11]. Fiber preparations have been known and used in the meat products technology for several decades [6,12]. However, nowadays, more and more precise and stringent requirements are placed on them regarding their functional properties [13,14,15]. There are numerous studies on the application of fiber preparations to meat balls [16], beef patties [17] and low-fat semi-dry fermented sausages [18]. There have also been many meat products on the food market, such as sausages labeled as “high in fiber” and “source of fiber”. According to Regulation (EC) No 1924/2006 of the European Parliament and of the Council of 20 December 2006 on nutrition and health claims made on foods, a claim that a food is high in fiber, as well as any claim likely to have the same meaning for the consumer, may only be used if the product contains at least 6 g of fiber per 100 g or at least 3 g of fiber per 100 kcal [19]. It is important to increase consumer acceptance in relation to meat products enriched with dietary fiber [20]. For the success of such products, the decisive factors include among others their sensory qualities. Consumers expect products of increasingly higher quality but at the same time at a price comparable to traditional products, i.e., without the addition of fiber.

One of the most important quality characteristics of both raw materials and food products, including meat and meat products, is color [21,22]. The perception of color significantly affects the positive or negative perception of the product by the consumer. The majority of consumers pay most attention to the external appearance of the product when shopping, and it is the color that is evaluated first [23,24]. In the case of cured meat products, the nitrosyl hemochromogen with its characteristic pink-red color is responsible for the color. The durability and stability of the color of cured products depends on many factors that are related to, among others, raw material, recipe (including the presence of additives), thermal treatment conditions, type of packaging and storage conditions [25]. The color of meat products will therefore also be conditioned by the addition of substances such as dietary fiber. The change in color of meat products caused by the introduction of various additives into their recipe may not be accepted by the consumers [2].

There are no data in the literature on the effect of the addition of fiber preparations on the quality of medium-grounded pasteurized and sterilized model canned meat products. Their thermal processing conditions are different compared to, for example, sausages or smoked meats, and therefore, different effects on their color can be expected. Therefore, it was the purpose to determine the effect of an addition of barley, wheat and oat fiber preparations with different fiber lengths on the color of medium-grounded pasteurized and sterilized model canned meat products, in quantities allowing the product to be indicated as “high fiber” or “source of fiber”. Wheat and oat fibers were selected for the research, as they are commonly used in the meat industry in Poland. Barley fiber was selected in order to verify the possibility of producing consumer-acceptable canned meat products containing β-glucan. This type of products are often consumed in Poland and could constitute a valuable additional source of fiber in the human diet.

## 2. Results and Discussion

With respect to the color, the fiber preparations used in this study can be divided into two groups. The first one included the barley dietary fiber preparation BG 300 with light brown color, the second one consisted of the remaining preparations, i.e., both wheat (WF 200R and WF 600R) and oat preparations (HF 200 and HF 600) with white color. The mean values of the color component L*, i.e., the color lightness of the tested dry preparations ranged from 77.6 (BG 300 preparation) to 93.1 units (WF 600R preparation; Table 1). The barley fiber preparation BG 300 was characterized by significantly the lowest (*p* ≤ 0.05) value of color component L*, which means it was the darkest. This was probably due to the presence of β-glucan in it [2]. There were no significant differences (*p* > 0.05) in color lightness between the dry preparations WF 200R, WF 600R, HF 200 and HF 600. Hydration of the fiber preparations, regardless of whether they underwent heat treatment (pasteurization or sterilization) or not, contributed to a significant (*p* ≤ 0.05) decrease in the value of the L* color component. This was most likely due to their absorption of water, which resulted in their darkening. The method of thermal treatment did not affect the values of color component L* and thus the lightness of all tested fiber preparations (Table 1). Both after hydration and after thermal treatment, BG 300 preparation was characterized by significantly (*p* ≤ 0.05) the lowest values of the L* color component in comparison with the other analyzed preparations.

The mean values of the a* color component, i.e., the share of red color of the tested dry fiber preparations, ranged from −0.02 (preparation HF 200) to 4.1 units (preparation BG 300), while the b* color component, i.e., the share of yellow color, ranged from 3.7 (preparation WF 600 R) to 15.7 units (preparation BG 300; Table 1). The barley fiber preparation BG 300 was characterized by the highest (*p* ≤ 0.05) values of color components a* and b*. Hydration and thermal treatment of the preparations caused differences in the values of those color components; however, the observed tendencies were not unequivocal.

Barley fiber preparation BG 300, containing significant amounts of β-glucan (>23%, manufacturer’s data), was characterized by a significantly (*p* ≤ 0.05) darker color and higher values of color components a* and b*, compared to the remaining wheat (WF 200R and WF 600R) and oat fiber preparations (HF 200 and HF 600), regardless of hydration and thermal treatment. The results obtained correspond to the observations made on the comparison of the color (instrumental measurement and sensory evaluation) of β-glucan concentrates (barley and oat) and wheat fiber preparation WF 200 [26]. Moreover, Temelli [27], obtaining barley concentrate of β-glucan (content of pure compound at the level of 76–86%) described the color of such preparation as light brown.

The mean values of Chroma (C*) of the tested dry fiber preparations ranged from 3.8 (WF 600R preparation) to 16.2 units (BG 300 preparation), while the hue angle (h*) from 75.4 (BG 300 preparation) to 92.5 units (HF 600 preparation; Table 1). Significantly the highest (*p* ≤ 0.05) value of C* and the lowest value of h* were found in BG 300 barley fiber preparation. Both after hydration and after thermal treatment, BG 300 preparation was characterized by significantly (*p* ≤ 0.05) the highest values of C* and the lowest values of the h* in comparison with the other analyzed preparations. Hydration and thermal treatment of the different fiber preparations caused differences in the values of those color components; however, the observed tendencies were not unequivocal. According to Gagaoua et al. [28] larger Chroma (C*) values indicate greater red color intensity. On the other hand larger hue angle (h*) values indicate a less red and more discolored lean.

Visual evaluation of the color of 10% suspensions of the tested fiber preparations before thermal treatment and after pasteurization or sterilization confirmed the results obtained in instrumental measurements (data not shown). The darkest (light brown) color, among all tested suspensions of the preparations, was found in the case of the barley fiber preparation BG 300. The color of suspensions of wheat fiber preparations WF 600R and WF 200R was defined as milky-white, whereas the color of suspensions of oat fiber preparations HF 200 and HF 600 was defined as milky-white with a delicate beige shade. It was evaluated as slightly darker in comparison with suspensions of wheat fiber preparations WF 200R and WF 600R, but definitely lighter in comparison with suspensions of barley fiber preparation BG 300. The visual changes of color of the suspensions tested caused by the applied thermal treatment (pasteurization or sterilization) were observed in no case.

The model medium-grounded pasteurized and sterilized canned meat products were characterized by water content of 70.6–74.7%, protein content of 12.6–13.8%, collagen content of 1.4–1.9%, fat content of 8.2–9.6%, and NaCl content of 1.9–2.0% (data not shown). There were no significant differences in the content of individual chemical components in the tested model canned meat products resulting from the addition of the fiber preparations. The differences observed resulted probably from natural differences in the chemical composition of the meat and fat raw materials used in the individual experimental batches.

Pasteurized canned meats were characterized by a color lightness (color component L*) ranging from 65.3 (canned products made with BG 300 at 6%) to 69.8 units (canned products made with HF 200 at 6%), color component a* values ranging from 6.2 (HF 600 6%) to 8.2 (BG 300 3%) and color component b* values ranging from 5.5 (control canned products) to 11.8 (BG 300 6%; Table 2). The sterilized canned meat products were characterized by the values of color component L* ranging from 63.6 (BG 300 6%) to 68.9 (WF 200 6%), color component a* from 6.3 (WF 200 6%) to 9.4 (BG 300 6%) and color component b* from 6.8 (control canned products) to 14.7 (BG 300 6%; Table 2). The addition of the barley fiber preparation BG 300 to the canned meat products, both at the level of 3 and 6%, caused a significant (*p* ≤ 0.05) darkening of the color of the products in comparison with the control products and the products with the addition of the other fiber preparations. These differences were also found regardless of the thermal treatment applied (Table 2).

Canned meat products containing an addition of barley fiber preparation BG 300, irrespective of the dose of preparation used (3 or 6%) and irrespective of the thermal treatment method (pasteurization or sterilization), showed a significant (*p* ≤ 0.05) increase in the b* color component in comparison with control canned meat products and those containing an addition of WF 200R and WF 600R as well as HF 200 and HF 600.

The majority of canned meat products produced with wheat (WF 200R and WF 600R) and oat fiber preparations (HF 200 and HF 600) showed a significant (*p* ≤ 0.05) increase in the proportion of yellow color (color component b*) in comparison with control canned meat products and those containing the BG 300 preparation. Irrespective of the type of preparation added, sterilized canned meat products showed significantly higher (*p* ≤ 0.05) values of color component b* in comparison with pasteurized canned meat products (Table 2). Moreover, in most cases, a significant (*p* ≤ 0.05) effect of increasing the addition of barley BG 300, wheat WF 200R and WF 600R or oat fiber preparations HF200 and HF600 from 3 to 6% on the values of color component b* of canned products subjected to the same thermal treatment was observed. The canned meat products with 6% addition of the fiber preparations were characterized by significantly (*p* ≤ 0.05) higher values of the color component b* (Table 2).

It should also be emphasized that irrespective of the amount of addition and method of thermal treatment, there was no effect (*p* > 0.05) of fiber length of wheat fiber (WF 200R and WF 600R) and barley fiber (HF 200 and HF 600) on the color components of the model canned meat products (Table 2). This is most likely due to the fact that no differences were also observed in the L*, a* and b* color components of the dry and hydrated preparations. No effect of wheat fiber length (WF 200R and WF 600R) regardless of the amount of fiber preparation addition (3 or 6%) on the color components of model homogenized sterilized canned meat products was also observed by Słowiński et al. [29].

Pasteurized canned meats were characterized by a Chroma (C*) values ranging from 9.4 (canned products made with WF 600R at 3%) to 14.3 units (canned products made with BG 300 at 6%), with h* values from 34.5 (control canned products) to 55.5 units (BG 300 6%). In contrast, the sterilized canned meat products were characterized by C* values ranging from 9.8 (control canned products) to 17.4 units (BG 300 6%), and h* values from 43.8 (control canned products) to 57.4 units (BG 300 6%; Table 2).

The addition of the barley fiber preparation BG 300 to the canned meat products, both at the level of 3 and 6%, resulted in a significant increase (*p* ≤ 0.05) in the C* values in comparison with the control products and the products with the addition of the other fiber preparations. These differences were observed regardless of the thermal treatment applied (Table 2). The canned meats produced with the fiber preparations were characterized by a significantly (*p* ≤ 0.05) higher h* values compared to the control product (Table 2). Their color became more yellow, which may not be fully acceptable by the consumers. Moreover, irrespective of the type of preparation added, sterilized canned meat products showed significantly higher (*p* ≤ 0.05) h* values in comparison with pasteurized canned meat products (Table 2). Moreover, in most cases, the canned meat products with 6% addition of the fiber preparations were characterized by significantly (*p* ≤ 0.05) higher values of this color component compared with products with 3% addition of fiber preparations (Table 2).

Table 3 presents the results of the absolute color difference (ΔE) between the canned products containing the tested fiber preparations and the control products. The impression of a different color (ΔE > 5) in comparison with the color of control canned meat products was obtained by evaluating the color of sterilized canned meat products with 3% addition of barley fiber BG 300 and pasteurized and sterilized canned meat products containing 6% addition of barley fiber preparation BG 300. On the other hand, differences in color visible to an inexperienced observer (ΔE ranging from 2 to 3.5) were found for almost all canned meat products containing a 6% addition of the wheat fiber preparations WF 200R and WF 600R and oat fiber preparations HF 200 and HF 600 (irrespective of the thermal treatment applied) and for pasteurized canned meat products with BG 300 fiber added at a level of 3%. Therefore, taking into account the color components described in the CIEL*a*b* scale and the absolute color difference ΔE calculated on their basis, it can be concluded that in the case of canned meat products with the addition of fiber preparation BG 300, the differences in color observed resulted mainly from the darkening of the blocks of canned meat with an increase in the proportion of yellow color.

It should also be noted that both in the case of stronger heating of the product as a result of the sterilization process and a higher addition of fiber preparation BG 300, intensification of color changes occurred (ΔE > 6, Table 3), which caused browning and darkening of blocks of canned meat products containing this preparation, visible to the naked eye. The causes of this phenomenon can be sought in the course of Maillard’s reactions, which are favored by both the high temperature of the sterilization process and the content of simple carbohydrates, with the simultaneous presence of compounds of protein character [30].

The possible darkening and browning of meat products caused by the addition of oat β-glucan concentrate was demonstrated by Alvarez and Barbut [31], who added Viscofiber^®^ preparation obtained from oats in the form of gel at three different concentrations of 0.15%, 0.3% and 0.6% to model meat batters. Moreover, Morin et al. [32] found that the use of barley β-glucan concentrate in the production of low-fat breakfast sausages caused their darkening and an increase in the values of color components a* and b*. In this case, the authors simultaneously reported a positive perception of the color change in the consumer evaluation of the sausages, which was probably due to the association of the observed color change with the reduction of fat content in them. In addition, Petersson et al. [33] observed significant color changes in low-fat sausages and fried beef pullets with an addition of barley β-glucan concentrate. The color changes were accompanied by higher scores awarded in the sensory evaluation. In contrast, the study by Trout et al. [34] showed that the addition of both potato starch, polydextrose and oat and pea fiber preparations did not affect the color changes of low-fat meatballs.

## 3. Materials and Methods

### 3.1. Research Material and Experimental Scheme

The first stage of the study involved color characterization of Vitacel^®^ fiber preparations (Rettenmaier & Söhne GmbH + Co. KG/Rosenberg, Germany), i.e., barley BG 300, wheat fiber preparations with different fiber lengths WF 200 R-250 µm and WF 600 R-80 µm, oat fiber preparations with different fiber lengths HF 200–250 µm and HF 600–80 µm by measuring the L*, a* and b* color components (the measure conditions are described below) of dry preparations and their 10% water suspensions: untreated by heat treatment and after pasteurization or sterilization processes. The color components L*, a* and b* were measured on a 1 cm layer of dry fiber preparation or its 10% suspension (20 g of the preparation were moistened with 180 g of water at 20 ± 1 °C, and then manually stirred to obtain homogeneous suspension) placed in a Petri dish. In addition C* (Chroma) and h* (hue angle) color components were calculated. The thermal treatment of the 10% suspension of the fiber preparations was carried out analogously to the model medium-grounded pasteurized and sterilized canned meat products (description below). The blocks of hydrated and gelled preparations were removed from the cans and sliced, and the color components L*, a* and b* were measured. Similar to the dry preparations, C* (Chroma) and h* (hue angle) color components were calculated.

The second stage of the study aimed to determine the effect of the type of fiber preparations (BG 300, WF 200R and WF 600R, HF 200 and HF 600) and the amount of their addition (3 or 6%) on the color of model medium-grounded pasteurized or sterilized canned meat products. The model medium-grounded pasteurized or sterilized canned meat products were made of cooled (4 ± 1 °C) chicken thigh muscles (85%) and pork jowl (15%). An addition of water/ice (45%) was applied to the weight of meat and fat raw materials, while an addition of a curing mixture (99.4% of NaCl and 0.6% of NaNO_2_; in amount of 1.8%), sodium isoascorbate (0.05%), poliphosphate preparation (0.3%; Tari P31; BK Giulini, Ladenburg, Germany) and spices (mixture of black pepper and herbal pepper in the ratio 1:2, in the amount of 0.3%; Kamis, Wólka Kosowska, Poland) was applied to the meat batter weight. The meat batters prepared in this way formed the basis for the production of experimental model medium-grounded pasteurized or sterilized canned meat products. The control model canned meat products did not contain added fiber, while the others contained a varied (3 or 6%) addition of a selected Vitacel^®^ fiber preparation.

**Figure 1:**
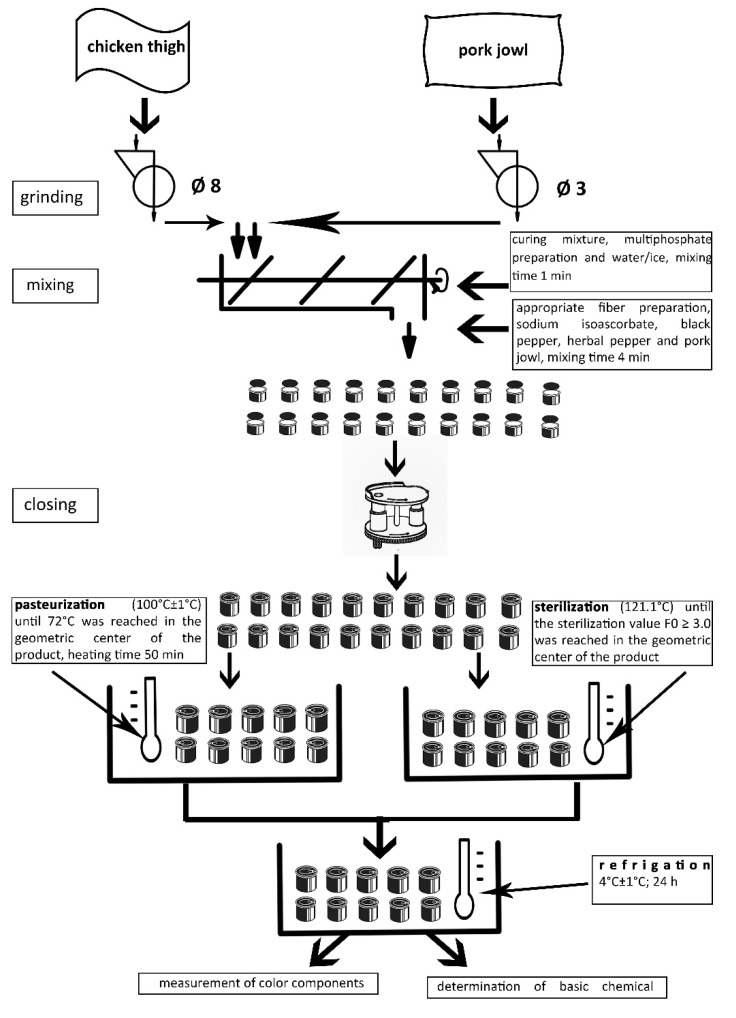
Scheme of the production process of medium-grounded pasteurized and sterilized model canned meat products.

Meat and fat raw materials, which were used to produce meat batters of model canned meat products were ground in a Mesko WN60 laboratory grinder (Mesko AL. 2–4, MESKO-AGD Sp. z o.o., Skarżysko-Kamienna, Poland). Chicken thigh muscles were ground using a mesh with a hole diameter of 8 mm, while pork jowls were ground using a mesh with a hole diameter of 3 mm. The mixing process was carried out in a Kenwood KM 070 laboratory mixer (Kenwood Ltd., Birmingham, UK), using a frame mixer. First, chicken thigh muscles were mixed with curing mixture, poliphosphate preparation and water/ice. The mixing time was about 1 min. In the next step, the appropriate fiber preparation, sodium isoascorbate, black pepper, herbal pepper and pork jowl were added. The mixing time was about 4 min, and the final temperature of the meat batter did not exceed 12 °C. Metal cans of 76 mm in diameter and 56 mm in height were filled with the 190 ± 0.1 g of meat batter prepared in this way, then closed with a “double overlap” using a semi-automatic closing machine (Nov-Handy Novopacké, Wahlstedt, Germany). For each product, 10 cans were prepared. The canned meat products prepared in this way were left to cure for approximately 6 h under refrigerated conditions (4 ± 1 °C). After this time, the cans underwent a pasteurization or sterilization process. The pasteurization process was carried out in a water bath (100 ± 1 °C) until 72 °C was reached in the geometric center of the product (the cans were heated for about 50 min, counting the time from the moment the water in the bath reached boiling point). To control the parameters of the pasteurization process in each production series, in one can a hole was made in the middle of its height and a special feedthrough was placed inside, enabling installation of sensors (TrackSense^®^ PRO Logger, Ellab, Hilleroed, Denmark). The sensors recorded the temperature inside the water bath and in the geometric center of the can every 30 s, with an accuracy of 0.1 °C. In turn, the sterilization process was carried out as described by Słowiński et al. [29]. After pasteurization or sterilization, the canned products were cooled in an ice-water bath, their surface was dried with a paper towel and then stored under refrigeration (4 ± 1°C) for 24 h. A simplified scheme of the production process of canned meat products is shown in Figure 1. The whole experiment was carried out three times in three independent production series.

### 3.2. Methods

#### 3.2.1. Measurement of Color Components

Color components were measured in the CIEL*a*b* scale using a Konica Minolta Chroma Meter CR-400 (Minolta, Osaka, Japan light source D65, observer angle 10°, with a measuring head hole of 8 mm, calibrated on a white standard L* 99.18; a* −0.07; b* −0.05). Measurements were made on dry preparations, their hydrated suspensions, and suspensions after thermal treatment. In addition, for canned meat products, color measurements were also performed on the freshly cut surface of the canned meat block. For each meat product in can, in each production series, each measurement was performed 5 times on 3 samples taken from 3 different cans and the mean value was used as the final result. In addition, Chroma (C*) and hue angle (h*) were calculated using the equations presented in AMSA [35].

In the present study, an additional criterion adopted by the International Commission on Illumination, the so-called absolute color difference (ΔE) was used to evaluate changes in color resulting from the addition of the tested Vitacel^®^ fiber preparations (barley BG 300, wheat WF 200R and WF 600R, oat HF 200 and HF 600) and the applied thermal treatment. It gives the possibility to perceive differences between two products and is defined according to the perception of colors by the human eye. It is assumed that the value of the absolute difference in color ΔE within the range of 0–2 indicates the inability to visually recognize the difference with the human visual sense, values between 2 and 3.5 allow an inexperienced observer to recognize the difference, values above 3.5 indicate a clear difference in color, while values above 5—the observer has the impression of two different colors [36].

#### 3.2.2. Content of the Basic Chemical Components

Determination of water, protein, fat, collagen and salt content was done by the method of near-infrared reflectance transmission (NIT) using calibration on artificial neural networks (ANN) in a FoodScan device (FOSS Analytical, Warsaw, Poland) and according to PN-A-82109 [37]. Each sample of model canned meat was previously ground according to AOAC 983.18 [38]. Then, approximately 180 g of the sample at 20 °C was placed in a glass circular cup with the dimensions (D: 140 mm, H: 17.5 mm). The prepared sample was then placed inside the device for the analysis. The analyses were determined on 3 samples taken from 3 different cans for each model meat product in can in each of three production series and the mean value was taken as a result.

#### 3.2.3. Statistical Analysis

The obtained results were statistically analyzed using Statistica ver. 12 PL (StatSoft, Inc., Tulsa, OK, USA). One-way analysis of variance (One-Way ANOVA) was used to determine the significance of differences between the mean values of the color components of the fiber preparations and the model medium-grounded pasteurized and sterilized canned meat products. The significance of differences between the canned products was verified using Tukey’s HSD test, with a significance level of α = 0.05.

## 4. Conclusions

Determination of the influence of the addition of fiber preparations on the color of meat products is not unequivocal and depends on many factors, such as the type of fiber preparation and its initial color, the amount of its addition, the share and arrangement of raw materials in the recipe of the designed meat product, the type and parameters of thermal treatment used. The fiber preparations used in this study can be divided into two groups with respect to color. The first group consisted of barley dietary fiber BG 300 of light brown color, the second group consisted of the remaining preparations, i.e., wheat (WF 200R and WF 600R) and oat dietary fibers (HF 200 and HF 600) of white color. The light brown color of BG 300 is due to the presence of β-glucan in it. This compound is nutritionally beneficial but also affects the color of the preparation. The addition of barley fiber BG 300 to model medium-grounded pasteurized or sterilized canned meat products caused, in comparison with the other canned products, a significant darkening and an increase in the proportion of yellow color. In industrial practice, this may result in poorer consumer acceptance of the meat product. The fiber length of wheat and barley fibers had no effect on the color components of the model medium-grounded canned meat products. The applied 6% addition of wheat fiber preparations WF 200R and WF 600R or oat fiber preparations HF 200 and HF 600 caused an impression of lightening of the color of canned meat products (ΔE > 2) in comparison with the control product.

## Figures and Tables

**Table 1:** L*, a* and b*, C* (Chroma) and h* (hue angle) color components of fiber preparations (mean ± standard deviation).

Characteristic	Color Component	Fiber Preparation					SEM
		BG 300	WF 200R	WF 600R	HF 200	HF 600	
Dry preparation	L*	77.6 ^aB^ ± 0.2	92.8 ^bB^ ± 0.5	93.1 ^bB^ ± 0.1	92.8 ^bB^ ± 0.4	92.7 ^bB^ ± 1.5	3.1
	a*	4.1 ^eB^ ± 0.1	0.2 ^dC^ ± 0.1	0.3 ^cB^ ± 0.1	−0.02 ^bB^ ± 0.1	−0.3 ^aA^ ± 0.1	0.8
	b*	15.7 ^eC^ ± 0.2	4.7 ^bC^ ± 0.1	3.8 ^aB^ ± 0.1	5.4 ^cC^ ± 0.1	7.0 ^dA^ ± 0.2	2.2
	C*	16.2 ^dB^ ± 0.2	4.7 ^bC^ ± 0.1	3.8 ^aB^ ± 0.1	5.4 ^bC^ ± 0.1	7.0 ^cA^ ± 0.1	2.3
	h*	75.4 ^aD^ ± 0.2	87.6 ^bA^ ± 0.1	85.5 ^bB^ ± 0.1	90.2 ^cA^ ± 0.1	92.5 ^cC^ ± 0.4	3.0
10% suspension before thermal treatment	L*	56.3 ^aA^ ± 0.8	69.6 ^bA^ ± 1.0	69.4 ^bA^ ± 1.5	67.5 ^bA^ ± 1.2	66.7 ^bA^ ± 1.7	2.5
	a*	3.1 ^cA^ ± 0.2	−0.1 ^aB^ ± 0.1	0.5 ^bC^ ± 0.1	−0.1 ^aA^ ± 0.1	−0.2 ^aA^ ± 0.3	0.6
	b*	10.0 ^cA^ ± 0.4	3.7 ^aA^ ± 0.2	3.4 ^aA^ ± 0.2	3.9 ^aA^ ± 0.2	6.9 ^bA^ ± 0.5	1.3
	C*	10.5 ^cA^ ± 0.2	3.7 ^aA^ ± 0.1	3.4 ^aA^ ± 0.1	3.9 ^aA^ ± 0.1	6.9 ^bA^ ± 0.1	1.4
	h*	72.8 ^aC^ ± 0.3	91.5 ^cB^ ± 0.2	81.6 ^bA^ ± 0.1	91.5 ^cB^ ± 0.3	91.7 ^cB^ ± 0.2	3.8
10% suspension after pasteurization	L*	54.7 ^aA^ ± 0.7	70.6 ^dA^ ± 0.7	70.0 ^dA^ ± 0.4	66.5 ^bA^ ± 0.6	68.4 ^cA^ ± 0.7	2.9
	a*	5.0 ^cC^ ± 0.1	−0.4 ^aA^ ± 0.1	−0.3 ^aA^ ± 0.1	−0.2 ^bA^ ± 0.1	−0.2 ^bA^ ± 0.1	1.1
	b*	10.1 ^cA^ ± 0.9	4.2 ^aB^ ± 0.2	4.0 ^aB^ ± 0.1	4.9 ^aB^ ± 0.2	7.9 ^bB^ ± 0.2	1.2
	C*	11.3 ^cA^ ± 0.4	4.2 ^aB^ ± 0.2	4.0 ^aB^ ± 0.1	4.9 ^aB^ ± 0.4	7.9 ^bB^ ± 0.1	1.4
	h*	63.7 ^aA^ ± 0.5	95.4 ^cD^ ± 0.1	94.3 ^cC^ ± 0.1	92.3 ^bC^ ± 0.1	91.5 ^bB^ ± 0.1	6.0
10% suspension after sterilization	L*	54.9 ^aA^ ± 0.6	70.6 ^cA^ ± 0.3	70.0 ^cA^ ± 0.9	67.4 ^bA^ ± 0.3	67.5 ^bA^ ± 0.6	2.9
	a*	5.6 ^cD^ ± 0.2	−0.3 ^bA^ ± 0.1	−0.2 ^bA^ ± 0.1	−0.1 ^abA^ ± 0.1	−0.02 ^aB^ ± 0.1	1.2
	b*	14.1 ^eB^ ± 0.1	4.6 ^bC^ ± 0.1	4.3 ^aC^ ± 0.1	5.6 ^cC^ ± 0.1	7.8 ^dB^ ± 0.2	1.8
	C*	15.2 ^dB^ ± 0.1	4.6 ^aC^ ± 0.1	4.3 ^aC^ ± 0.1	5.6 ^bC^ ± 0.1	7.8 ^cB^ ± 0.1	2.0
	h*	68.3 ^aB^ ± 0.1	93.7 ^cC^ ± 0.1	92.7 ^bC^ ± 0.1	91.0 ^bB^ ± 0.1	90.1 ^bA^ ± 0.2	4.8
SEM	L*	5.6	5.6	5.8	6.4	6.3	
	a*	0.5	0.1	0.2	0.04	0.1	
	b*	1.4	0.2	0.2	0.4	0.3	
	C*	1.4	0.2	0.2	0.4	0.3	
	h*	2.6	1.7	3.0	0.4	0.5	

^a–e^—Means in the row marked with different letters are significantly different (*p* ≤ 0.05) influence of the type of fiber preparation. ^A–D^—Means in the column, for appropriate color component, marked with different letters are significantly different (*p* ≤ 0.05). L*, a*, b*, C*, h*—Color components: lightness, redness, yellowness, Chroma, hue angle. SEM—Standard error of mean.

**Table 2:** L*, a* and b*, C* (Chroma) and h* (hue angle) color components of model medium-grounded pasteurized and sterilized model canned meat products (mean ± standard deviation).

Color Component		Control Canned Meat Products ^1^	The Amount of Fiber Preparation Added	Method of Thermal Treatment	Canned Meat Products with Fiber Preparations					SEM
					BG 300	WF 200R	WF 600R	HF 200	HF 600	
L*		67.3 ^bA^ ± 1.0	3%	Pasteurization	65.7 ^aA^ ± 0.1	68.2 ^bA^ ± 0.7	67.6 ^bA^ ± 0.6	68.6 ^bA^ ± 0.4	66.7 ^bA^ ± 0.7	0.4
a*		8.0 ^aA^ ± 0.6			8.2 ^aA^ ± 0.8	7.5 ^aA^ ± 0.6	6.5 ^aA^ ± 0.7	7.1 ^aA^ ± 0.5	7.3 ^aA^ ± 0.7	0.3
b*		5.5 ^aA^ ± 0.2			8.2 ^cA^* ± 0.5	6.7 ^bA^* ± 0.2	6.8 ^bA^* ± 0.3	6.4 ^bA^* ± 0.5	6.8 ^bA^* ± 0.2	0.4
C*		9.7 ^aA^ ± 0.2			11.6 ^cA^* ± 0.5	10.1 ^bA^ ± 0.2	9.4 ^aA^ ± 0.2	9.6 ^aA^ ± 0.2	10.0 ^bA^ ± 0.2	0.3
h*		34.5 ^aA^ ± 0.3			45.0 ^dA^* ± 0.5	41.8 ^bA^* ± 0.3	46.3 ^dA^* ± 0.4	42.0 ^bA^* ± 0.2	43.0 ^cA^* ± 0.24	1.7
L*		66.9 ^bA^ ± 0.4		Sterilization	63.8 ^aB^ ± 0.5	67.4 ^bA^ ± 0.8	67.3 ^bA^ ± 1.0	67.8 ^bA^ ± 0.6	67.5 ^bA^ ± 0.9	0.6
a*		7.1 ^aA^ ± 0.6			9.3 ^bA^ ± 0.7	6.7 ^aA^ ± 0.9	7.0 ^aA^ ± 0.9	7.3 ^abA^ ± 0.4	7.1 ^abA^ ± 0.9	0.4
b*		6.8 ^aB^ ± 0.4			11.9 ^cB^* ± 0.3	8.1 ^bB^* ± 0.3	8.2 ^bB^ ± 0.6	8.0 ^bB^* ± 0.6	8.3 ^bB^* ± 0.4	0.7
C*		9.8 ^aA^ ± 0.3			15.1 ^cB^* ± 0.4	10.5 ^bA^ ± 0.4	10.8 ^bB^ ± 0.7	10.8 ^bB^* ± 0.4	10.9 ^bA^ ± 0.6	0.8
h*		43.8 ^aB^ ± 0.3			52.0 ^dB^* ± 0.5	50.4 ^cB^* ± 0.6	49.5 ^cB^ ± 0.8	47.6 ^bB^* ± 0.5	49.5 ^cB^* ± 0.6	1.2
L*		67.3 ^bA^ ± 1.0	6%	Pasteurization	65.3 ^aA^ ± 0.7	69.4 ^bA^ ± 0.9	68.5 ^bA^ ± 0.7	69.8 ^bA^ ± 0.6	67.8 ^bA^ ± 0.7	0.7
a*		8.0 ^aA^ ± 0.6			8.1 ^bA^ ± 0.8	6.5 ^aA^ ± 0.4	6.4 ^aA^ ± 0.7	6.4 ^aA^ ± 0.9	6.2 ^aA^ ± 0.5	0.4
b*		5.5 ^aA^ ± 0.2			11.8 ^cA^* ± 1.0	7.8 ^bA^* ± 0.4	7.4 ^bA^* ± 0.3	7.8 ^bA^* ± 0.5	8.2 ^bA^* ± 0.1	0.8
C*		9.7 ^aA^ ± 0.2			14.3 ^bA^* ± 0.5	10.2 ^aA^ ± 0.2	9.8 ^aA^ ± 0.3	10.1 ^aA^ ± 0.1	10.3 ^aA^ ± 0.2	0.7
h*		34.5 ^aA^ ± 0.3			55.5 ^dA^* ± 0.5	50.2 ^cA^* ± 0.2	49.1 ^bA^* ± 0.5	50.6 ^cA^* ± 0.6	52.9 ^cA^* ± 0.4	3.0
L*		66.9 ^bA^ ± 0.4		Sterilization	63.6 ^aB^ ± 0.2	68.9 ^bA^ ± 0.9	67.8 ^bA^ ± 0.4	68.5 ^bA^ ± 0.6	68.0^bA^ ± 0.6	0.8
a*		7.1 ^aA^ ± 0.6			9.4 ^bA^ ± 0.9	6.3 ^aA^ ± 0.6	7.2 ^aA^ ± 0.6	7.5 ^abA^ ± 0.9	6.9 ^aA^ ± 0.6	0.4
b*		6.8 ^aB^ ± 0.4			14.7 ^cB^* ± 1.0	9.2 ^bB^* ± 0.2	8.3 ^bA^ ± 0.6	9.3 ^bB^* ± 0.6	9.6 ^bB^* ± 0.5	1.1
C*		9.8 ^aA^ ± 0.3			17.4 ^cB^* ± 0.5	11.2 ^bB^ ± 0.3	11.0 ^bB^ ± 0.3	11.9 ^bB^* ± 0.2	11.8 ^bB^ ± 0.4	1.1
h*		43.8 ^aB^ ± 0.3			57.4 ^dB^* ± 0.5	55.6 ^cB^* ± 0.4	49.1 ^bA^ ± 0.4	51.1 ^bA^* ± 0.6	54.3 ^cB^* ± 0.6	2.0
SEM	L*	0.2			0.5	0.4	0.3	0.4	0.3	
	a*	0.5			0.3	0.3	0.2	0.2	0.2	
	b*	0.6			1.3	0.5	0.4	0.6	0.6	
	C*	0.1			1.2	0.2	0.4	0.5	0.4	
	h*	4.6			2.7	2.9	0.7	2.1	2.5	

^1^ The results for control canned meat products were included in both analyses for 3 and 6% of fiber preparation addition levels. ^a–d^—Means in the row marked with different letters are significantly different. (*p* ≤ 0.05)—Influence of the type of fiber preparation. ^A,B^—Means in the column, for appropriate color component and the same dose of fiber preparation, marked with different letters are significantly different. (*p* ≤ 0.05)—Influence of the thermal treatment method. *—Means in the column, for appropriate color component and the same thermal treatment method, marked with * are significantly different. (*p* ≤ 0.05)—The influence of dose of fiber preparation. L*, a*, b*, C*, h*—Color components: lightness, redness, yellowness, Chroma, hue angle. SEM—Standard error of mean.

**Table 3:** Absolute color difference (ΔE).

The Amount of Fiber Preparation Added	Method of Thermal Treatment	Canned Meat Products with Fiber Preparations					SEM
		BG 300	WF 200R	WF 600R	HF 200	HF 600	
3%	Pasteurization	3.16	1.58	2.05	1.78	1.56	0.3
	Sterilization	6.34	1.49	1.50	1.53	1.65	1.0
6%	Pasteurization	6.66	3.50	2.73	3.74	3.35	0.7
	Sterilization	8.93	3.21	1.54	3.09	3.09	1.3
SEM		1.2	0.5	0.3	0.5	0.5	

ΔE calculated in relation to the control canned meat products. SEM—Standard error of mean.

## Sample Availability

Samples of the compounds are not available from the authors.
